# Fermentation quality, bacterial community, and aerobic stability of ensiling *Leymus chinensis* with lactic acid bacteria or/and water after long-term storage

**DOI:** 10.3389/fmicb.2022.959018

**Published:** 2022-10-18

**Authors:** Haiwen Xu, Yanlin Xue, Na Na, Nier Wu, Yi Zhao, Lin Sun, Moge Qili, Tianwei Wang, Jin Zhong

**Affiliations:** ^1^College of Foreign Languages, Inner Mongolia University of Finance and Economics, Hohhot, China; ^2^Inner Mongolia Key Laboratory of Microbial Ecology of Silage, Inner Mongolia Engineering Research Center of Development and Utilization of Microbial Resources in Silage, Inner Mongolia Academy of Agriculture and Animal Husbandry Science, Hohhot, China; ^3^Institute of Microbiology, Chinese Academy of Sciences, Beijing, China

**Keywords:** bacterial diversity, *Leymus chinensis* silage, microbial counts, nutritional compositions, silage temperature

## Abstract

*Leymus chinensis* is a major forage resource for herbivores on typical steppe and meadow steppes in Northern China. This study aimed to reveal the fermentation quality, bacterial community, and aerobic stability of *L. chinensis* silage treated with lactic acid bacteria or/and water after long-term storage. *Leymus chinensis* was harvested at the heading stage and ensiled with lactic acid bacteria [LAB, 2.00 ml/kg fresh weight (FW) of LAB, L], water (100 ml/kg FW of distilled water, W), or a combination of both [2.00 ml/kg fresh weight (FW) of LAB and 100 ml/kg FW of distilled water, LW] in polyethylene laboratory-scale silos (diameter, 20 cm; height, 30 cm) at a density of 650 kg/m^3^. As a control silage (CK), untreated *L. chinensis* silage was also assessed. The samples were taken at 0 day of opening after 300 days of ensiling (CK_0d, L_0d, W_0d, and LW_0d) and at 10 days of opening (CK_10d, L_10d, W_10d, and LW_10d). The fermentation quality, microbial counts, bacterial community, and aerobic stability of the silage were assessed. The CK_0d contained higher pH and aerobic bacteria count, and lower LA and BC concentrations than L_0d, W_0d, and LW_0d (*p* < 0.05), and the LAB and yeasts were only detected in CK at 0 day of opening. *Lactobacillus* had the most abundance among bacterial genera in all silages at 0 day of opening. Just CK had 2°C above the ambient temperature during aerobic exposure (at 224 h). During aerobic exposure, the pH and microbial counts in CK increased (*p* < 0.05), and *Lactobacillus* in L and LW had decreasing abundance (*p* < 0.05). The CK_10d had higher pH and microbial counts, and lower lactic acid and buffering capacity than L_10d, W_10d, and LW_10d (*p* < 0.05). At 10 days of opening, the coliforms and yeasts were just detected in CK, and *Lactobacillus* also had the most abundance among bacterial genera in all silages at 10 days of opening. Overall, inoculating LAB and adding water improved the fermentation quality and the aerobic exposure of *L. chinensis* silage after long-term storage. The activities of coliforms and yeasts during aerobic exposure contributed to the aerobic deterioration of *L. chinensis* silage without any treating. *Lactobacillus* dominated the bacterial communities of all silage at 0 and 10 days of opening. During aerobic exposure, the abundance of *Lactobacillus* reduced in *L. chinensis* silage treated with LAB or water.

## Introduction

*Leymus chinensis* is one of the dominating grasses, a major forage resource for herbivores, and mostly utilized for grazing and haymaking on typical steppe and meadow steppes in Northern China (Zhang et al., 2016; Du et al., 2020). Its quality, palatability, and productivity were affected by the seasonal changes (Kang et al., 2007), and it is difficult to provide high-quality *L. chinensis* year-round under grazing and haymaking systems (Li et al., 2022). Ensiling is a satisfactory method for preserving *L. chinensis* to overcome the above shortcomings (Pahlow et al., 2003; Xu et al., 2021). However, the insufficient epiphytic lactic acid bacteria (LAB) and the low moisture content (less than 600 g/kg) in materials negatively affect the fermentation process and the microbial dynamics and induce the *Enterobacteriaceae* succession in *L. chinensis* silage (Xue et al., 2017; Xu et al., 2021). Previous studies reported that inoculating LAB at ensiling can improve the fermentation quality, aerobic stability, and digestibility of *L. chinensis* silage (Tian et al., 2014; Zhang et al., 2015a; Zhang and Yu, 2017). Moreover, Xu et al. (2021) revealed that ensiling *L. chinensis* with LAB or/and water promotes the fermentation process and LAB succession during fermentation and improves the fermentation quality in the terminal silage, and *Enterobacteriaceae* dominates the bacterial community during late fermentation phase (from 35 to 60 days) in the silage treated with water.

The previous studies mainly focused on the characteristics of *L. chinensis* silage during short-term storage (less than 100 days). Those included the dynamics of bacterial community and fermentation quality from 0 to 60 days of ensiling (Xu et al., 2021), the effects of inoculating LAB, growing locations and stages, and chopping length on the fermentation quality or/and aerobic stability (Tian et al., 2014; Zhang et al., 2015a,b,c, 2017; Xue et al., 2017; Sun et al., 2020), and the identification of LAB isolated from *L. chinensis* silage (Zhang and Yu, 2017). It is very important to study the characteristics of silage after long-term storage (more than 200 days), because silage is a kind of roughage that can be supplied to the ruminants year-round. Previous studied reported the microbial communities, fermentation quality, nutrition compositions, and aerobic stability of whole-plant corn silage after 300 and 350 days of storage (Bai et al., 2021; Wang et al., 2021). Nevertheless, there are no reports of studies on the *L. chinensis* silage in this area. So, we hypothesized that there were differences in fermentation quality and microbial communities among *L. chinensis* silages treated with LAB or/and water after long-term storage. The objective of this study was to reveal the fermentation quality, bacterial community, and aerobic stability of ensiling *L. chinensis* with lactic acid bacteria or/and water at 300 days of storing.

## Materials and methods

### Silage preparation

*Leymus chinensis* was harvested at heading stage (Xue et al., 2017) from three sampling sites randomly selected as replicates on a commercial farm (in typical steppe, 116°29′37′′E, 44°13′10′′N, Inner Mongolia Caodu Grassland Husbandry Co., Ltd., Xilinhot, China) on 28 July 2019. The fresh forage from each site was separately chopped into 2- to 3-cm pieces using a chaff cutter (Hongguang Industry & Trade Co., Ltd., Zhejiang, China), mixed thoroughly, and divided into four batches (15 kg for each batch) for four treatments as follows: CK, 2.00 ml/kg fresh weight (FW) of distilled water; L, 2.00 g/t FW of LAB inoculant and 2.00 ml/kg FW of distilled water; W, 100 ml/kg FW of distilled water; LW, 2.00 g/t FW of LAB inoculant and 100 ml/kg FW of distilled water. There were three replications per treatment. The LAB inoculant was bought from Xinlaiwang Biotechnology Co., Ltd, Yangzhou, China, and its compositions were *Lactobacillus plantarum* (≥6 × 10^10^ colony-forming units (CFUs)/g) and *Lactobacillus casei* (≥4 × 10^10^ CFU/g). After mixing each treatment uniformly for all samples from each site, approximately 14 kg of each forage sample was packed into two polyethylene laboratory-scale silos (diameter, 20 cm; height, 30 cm; approximately 6.2 kg for each silo) at a density of 650 kg/m^3^. The 24 silos (4 treatments × 3 replicates × 2 opening time) were stored at ambient temperature (22–25°C) and sampled at 300 days of storage to determine the aerobic stability, fermentation quality, microbial counts, bacterial community, and nutritional components.

### Sampling and aerobic stability assessment

The silos were opened after 300 days of ensiling. For each sampling site per treatment (two silos), one silo selected randomly was sampled at 0 day of opening (CK_0d, L_0d, W_0d, and LW_0d), and the other silo was used to assess the aerobic stability (silage temperature) according to Sun et al. (2020) and Wang et al. (2020) and sampled at 10 days of opening (CK_10d, L_10d, W_10d, and LW_10d). Those samples were used to measure the fermentation quality, microbial counts, bacterial community, and nutritional components of silage. The silage temperature and the ambient temperature were measured by an inserting SMOWO Multi-Channel Data Logger (MDL-1048A; Shanghai Tianhe Automation Instrument Co., Ltd., Shanghai, China).

### Fermentation quality

The silage samples (approximately 500 g per sample) were dried in a forced-air oven (BPG-9240A, Shanghai Yiheng Scientific Instrument Co., Ltd., Shanghai, China) for 48 h to measure the dry matter (DM) content and then ground through a 1-mm screen with a mill (FS-6D; Fichi Machinery Equipment Co., Ltd., Shandong, China) for measuring buffering capacity (BC) and nutritional components.

The fermentation quality was analyzed from silage extract. Fresh silage (25 g) and sterile water (225 ml) were homogenized for 100 s using a flap-type sterile homogenizer (JX-05, Shanghai Jingxin Industrial Development Co., Ltd., Shanghai, China) and filtered through four layers of sterile cheesecloth (Xu et al., 2021). The pH was measured by a pH meter (PB-10, Sartorius, Gottingen, Germany). The silage extract was filtrated through a filter membrane (0.22 μm) and then measured by a high-performance liquid chromatography (DAD, 210 nm, SPD-20A, Shimadzu Co., Ltd., Kyoto, Japan) to assess the organic acids [lactic, acetic, propionic, and butyric acid (LA, AA PA, and BA)] concentrations in silage. The conditions were as follows: detector, SPD-20A diode array detector, 210 nm; column, Shodex RS Pak KC-811, 50°C (Showa Denko K.K., Kawasaki, Japan); mobile phase, 3 mM HClO_4_, 1.0 ml/min (Wang et al., 2021).

The silage extract was measured by a Kjeltec autoanalyzer (8400; Foss Co., Ltd., Hillerød, Denmark) using the Kjeldahl method to assess the ammonia nitrogen (AN) concentration in silage (AOAC International, 2005). The powder sample was measured according to Playne and McDonald (1966) for assessing the buffering capacity (BC) in silage.

### Microbial counts and bacterial community

The microbial counts in fresh forage or silage were assessed according to Cai (1999). Coliforms, aerobic bacteria, and yeasts were cultured on violet red bile agar, nutrient agar, and potato dextrose agar, respectively, in an incubator (LRH-70, Shanghai Yiheng Science Instruments Co., Ltd., Shanghai, China) at 30°C for 72 h. Moreover, LAB were cultured on Man, Rogosa, and Sharpe agar under anaerobic condition in the same incubator at 30°C for 72 h.

The bacterial DNA in the silage was extracted using an E.Z.N.A. ^®^Stool DNA Kit (D4015, Omega Bio-tek, Inc., GA, USA) following the manufacturer’s instructions. The V3-V4 region of the bacterial rRNA gene was amplified by a polymerase chain reaction (PCR) with primers 341F (5′-CCTACGGGNGGCWGCAG-3′) and 805R (5′-GACTACHVGGGTATCTAATCC-3′). The conditions were as follows: 98°C for 30 s followed by 32 cycles of denaturation at 98°C for 10 s, annealing at 54°C for 30 s, and extension at 72°C for 45 s, followed by a final extension at 72°C for 10 min (Logue et al., 2016). The PCR products were purified by an AMPure XT beads (Beckman Coulter Genomics, Danvers, MA, USA) and then quantified by a Qubit (Invitrogen, USA). The purified and quantified PCR products were sequenced by an Illumina NovaSeq PE250 platform according to manufacturer’s recommendations, provided by LC-Bio (Hangzhou Lianchuan Biotechnology Co., Ltd., Hangzhou, China). The paired-end reads were merged using FLASH. Principal component analysis (PCA) and bacterial community differences between 0 and 10 days of opening for each treatment were analyzed using R 3.6.1. Sequencing data were submitted to the NCBI Sequence Read Archive database (accession number: PRJNA841435).

### Nutrition compositions

The total nitrogen (TN) in fresh forage or silage was detected by a Kjeltec autoanalyzer (8400; Foss Co., Ltd., Hillerød, Denmark) with copper as the catalyst according to the Kjeldahl method, and the TN multiplied by 6.25 was crude protein (CP) concentration in silage. The neutral detergent fiber (NDF) and acid detergent fiber (ADF) concentrations were assessed by an ANKOM fiber analyzer (ANKOM 2000, ANKOM Technology, Macedon, NY, USA) without heat-stable amylase according to the method of Van Soest et al. (1991). The ash concentration in silage was assessed according to AOAC International (2005).

### Statistical analyses

The differences between 2 opening times (0 and 10 days of opening) for each treatment (1) and among 4 treatments (CK, L, W, and LW) for each opening time (2) were analyzed with the GLM procedure of SAS (SAS System for Windows, version 9.1.3; SAS Institute Inc., Cary, NC, USA). The statistical model is as follows:


(1) $$X_{\text{i}}=\mu +\alpha_{\text{i}}+\varepsilon_{\text{i}}$$

where X_i_ is the observation, μ is the overall mean, α_i_ is the effect of opening time (*k* = 0 and 10 days of opening), and ε_i_ is the error.


(2) $$X_{\text{j}}=\mu +\beta_{\text{j}}+\varepsilon_{\text{j}}$$

where X_j_ is the observation, μ is the overall mean, β_j_ is the effect of treatments (j = CK, L, W, and LW), and ε_j_ is the error.

## Results

### Characteristics of fresh forage

The characteristics of *L. chinensis* before ensiling are presented in Table 1.

**TABLE 1:** The pH, microbial counts (log colony-forming units/g fresh weight), dry matter (DM, g/kg), nutritional compositions concentrations (g/kg DM), and buffering capacity (BC, mE/kg DM) in fresh *Leymus chinensis* (*n* = 3).

Items	pH	Microbial counts				DM	Nutritional compositions					BC
			
		LAB	Coliforms	Aerobic bacteria	Yeast		CP	WSC	NDF	ADF	Ash	
Fresh *L. chinensis*	5.95	3.49	6.12	6.74	4.17	487	9.88	6.23	356	184	54.9	218
SD	0.061	0.449	0.094	0.088	0.432	7.22	0.115	0.313	5.69	3.21	1.68	6.22

LAB, lactic acid bacteria; CP, crude protein; WSC, water soluble carbohydrates; NDF, neutral detergent fiber; ADF, acid detergent fiber. SD, standard deviation.

### Aerobic stability

**FIGURE 1:**
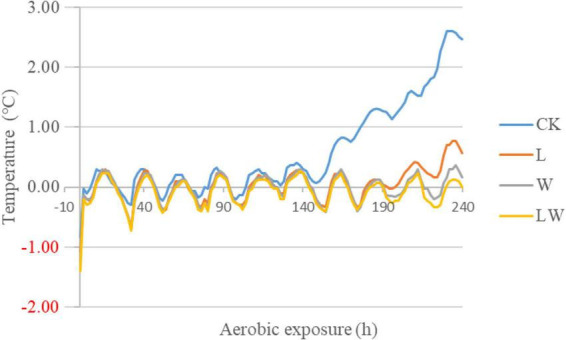
The degree of silage temperature (°C) above ambient temperature in *Leymus chinensis* silage during aerobic exposure (*n* = 3). CK, ensiling *L. chinensis* with 2.00 ml/kg fresh weight (FW) of distilled water; L, ensiling *L. chinensis* with 2.00 g/t FW of lactic acid bacteria (LAB) inoculant and 2.00 ml/kg FW of distilled water; W, ensiling *L. chinensis* with 100 ml/kg FW of distilled water; LW, ensiling *L. chinensis* with 2.00 g/t FW of LAB inoculant and 100.0 ml/kg FW of distilled water.

The temperature of CK began to rise at 150 h of opening and had 2°C above the ambient temperature at 224 h of opening (Figure 1). However, other treatments did not reach to 2°C above the ambient temperature during 10 days of opening.

### Fermentation quality

At 0 day of opening, the CK contained higher pH but lower LA and BC than other treatments (*p* < 0.01), the L contained lower LA and BC than W and LW (*p* < 0.01), and the W contained higher AN and BC than other treatments (*p* < 0.01) (Table 2). At 10 days after opening, the CK contained higher pH and lower LA and BC than other treatments (*p* < 0.01), the L contained higher pH and lower LA than W and LW (*p* < 0.01), the W contained higher pH than LW and higher BC than L (*p* < 0.01). The CK_0d had lower pH than CK_10d (*p* < 0.01), the L_0d contained higher LA and BC contents than L_10d (*p* < 0.01), the W_0d contained higher pH, AN, and BC than W_10d (*p* < 0.05), and LW_0d contained higher BC than LW_10d (*p* < 0.05). The inoculating LAB, adding water, and opening time had main effect on pH, LA, AN, and BC (*p* < 0.05), and the adding water and opening time had main effect on AA (*p* < 0.05).

**TABLE 2:** The pH, organic acid concentrations [g/kg dry matter (DM)], ammonia nitrogen/total nitrogen (AN, g/kg total nitrogen), and buffering capacity (BC, mE/kg DM) in *Leymus chinensis* silages (*n* = 3).

Items		CK	L	W	LW	SEM	*p*-value
pH	0 d	5.29Ba	3.96b	3.88Ab	3.72b	0.070	<0.001
	10 d	6.36Aa	3.88b	3.80Bc	3.68d	0.018	<0.001
	SEM	0.094	0.036	0.016	0.017		
	*p*-value	0.001	0.187	0.023	0.119		
LA	0 d	4.20c	14.8Ab	29.6a	24.8a	2.13	<0.001
	10 d	2.80c	9.33Bb	18.5a	20.2a	1.45	<0.001
	SEM	0.686	0.345	3.23	1.52		
	*p*-value	0.228	<0.001	0.071	0.096		
AA	0 d	14.7	9.90	16.5	19.2	2.84	0.205
	10 d	10.7	6.08	10.3	13.6	2.14	0.179
	SEM	1.875	1.131	3.93	2.25		
	*p*-value	0.204	0.077	0.324	0.151		
AN	0 d	1.45b	1.87b	5.91Aa	2.06b	0.311	<0.001
	10 d	1.84	1.71	2.42B	2.10	0.242	0.247
	SEM	0.232	0.407	0.295	0.064		
	*p*-value	0.308	0.799	0.001	0.681		
BC	0 d	307d	468Ac	508Aa	493Ab	2.30	<0.001
	10 d	265c	447Bb	488Ba	473Bab	9.02	<0.001
	SEM	11.6	3.21	4.05	3.50		
	*p*-value	0.062	0.009	0.026	0.017		

CK, ensiling *L. chinensis* with 2.00 m/kg fresh weight (FW) of distilled water; L, ensiling *L. chinensis* with 2.00 g/t FW of lactic acid bacteria (LAB) inoculant and 2.00 ml/kg FW of distilled water; W, ensiling *L. chinensis* with 100 mL/kg FW of distilled water; LW, ensiling *L. chinensis* with 2.00 g/t FW of LAB inoculant and 100.0 ml/kg FW of distilled water. LA, lactic acid; AA, acetic acid. Values with different lowercase letters (a, b, c, and d) indicate significant differences among treatments on the same day (*p* < 0.05). Values with different uppercase letters (A and B) indicate the significant differences between 0 and 10 days of opening for the same treatment (*p* < 0.05). SEM, standard error of the means.

### Microbial counts

At 0 day of opening, the LAB and yeasts were only detected in CK, with the highest aerobic bacteria count (*p* < 0.01); moreover, the L and LW contained lower aerobic bacteria count than other treatments (*p* < 0.01) (Table 3). At 10 days of opening, coliforms and yeasts were also only detected in CK, with the highest LAB and aerobic bacteria counts (*p* < 0.01); moreover, the aerobic bacteria count in LW was higher than that in L and lower than that in CK and W (*p* < 0.01). Furthermore, the CK_10d and LW_10d contained higher aerobic bacteria count than CK_0d and LW_0d, respectively (*p* < 0.05). The LAB, coliforms, aerobic bacteria, and yeasts counts were mainly affected by inoculating LAB, adding water, and opening time (*p* < 0.01).

**TABLE 3:** Microbial counts (log colony-forming units/g fresh weight) in *Leymus chinensis* silages (*n* = 3).

Items		CK	L	W	LW	SEM	*p*-value
LAB	0 d	3.81Ba	ND	ND	ND	0.045	<0.001
	10 d	6.55Aa	2.91Ab	2.75Ab	3.01Ab	0.135	<0.001
	SEM	0.150	0.049	0.044	0.071		
	*p*-value	<0.001	< 0.001	<0.001	<0.001		
Coliforms	0 d	ND	ND	ND	ND	–	–
	10 d	4.79Aa	ND	ND	ND	0.254	<0.001
	SEM	0.359	–	–	–		
	*p*-value	<0.001	–	–	–		
Aerobic bacteria	0 d	5.07Ba	3.39c	4.13b	3.54Bc	0.124	<0.001
	10 d	5.89Aa	3.53d	4.18b	3.76Ac	0.047	<0.001
	SEM	0.093	0.150	0.040	0.050		
	*p*-value	0.04	0.554	0.402	0.039		
Yeasts	0 d	5.25Ba	ND	ND	ND	0.062	<0.001
	10 d	6.19Aa	ND	ND	ND	0.066	<0.001
	SEM	0.128	–	–	–		
	*p*-value	0.007	–	–	–		

CK, ensiling *L. chinensis* with 2.00 ml/kg fresh weight (FW) of distilled water; L, ensiling *L. chinensis* with 2.00 g/t FW of lactic acid bacteria (LAB) inoculant and 2.00 ml/kg FW of distilled water; W, ensiling *L. chinensis* with 100 ml/kg FW of distilled water; LW, ensiling *L. chinensis* with 2.00 g/t FW of LAB inoculant and 100.0 ml/kg FW of distilled water. Values with different lowercase letters (a, b, c, and d) indicate significant differences among treatments on the same day (*p* < 0.05). Values with different uppercase letters (A and B) indicate the significant differences between 0 and 10 days of opening for the same treatment (*p* < 0.05). SEM, standard error of the means. LAB, lactic acid bacteria. ND, not detected.

### Bacterial diversity

At 0 day of opening, the LW contained the lower valid tags than CK and L (*p* < 0.05), and the L contained the lower Simpson index than other treatments (*p* < 0.01) (Table 4). At 10 days of opening, the CK and LW contained higher valid tags than L and W (*p* < 0.01), and the CK contained lower observed OTUs, and Shannon and Chao1 indexes than other treatments (*p* < 0.01). The L_10d contained lower valid tags and higher Shannon and Simpson indexes than L_0d (*p* < 0.05); in addition, W_10d had higher Shannon and Simpson indexes than W_0d (*p* < 0.05).

**TABLE 4:** Sequencing data and alpha diversity of bacteria in *Leymus chinensis* silages (*n* = 3).

Items		CK	L	W	LW	SEM	*p*-value
Raw tags	0 d	84166	85351	82691	84524	1196	0.499
	10 d	84098	79658	75764	83806	5066	0.630
	SEM	1222	3669	6069	1550		
	*p*-value	0.971	0.334	0.365	0.638		
Valid tags	0 d	79920a	81657Aa	69574ab	65639b	3025	0.014
	10 d	78094a	61773Bb	59659b	77947a	3290	0.006
	SEM	1402	1531	4542	3875		
	*p*-value	0.409	<0.001	0.405	0.201		
Observed OTUs	0 d	204	240	191	224	31.8	0.710
	10 d	125b	240a	237a	277a	21.8	0.006
	SEM	30.7	14.9	26.9	33.0		
	*p*-value	0.144	0.976	0.762	0.139		
Shannon	0 d	2.85	1.91B	3.45B	3.45	0.384	0.066
	10 d	2.47b	4.02Aa	4.78Aa	4.69a	0.347	0.005
	SEM	0.214	0.524	0.210	0.414		
	*p*-value	0.281	0.046	0.011	0.101		
Simpson	0 d	0.712a	0.358Bb	0.768Ba	0.736a	0.064	0.006
	10 d	0.651	0.764A	0.863A	0.833	0.057	0.124
	SEM	0.065	0.084	0.009	0.058		
	*p*-value	0.569	0.027	<0.001	0.482		
Chao1	0 d	212	254	201	229	34.2	0.728
	10 d	129b	246a	240a	281a	22.0	0.006
	SEM	32.5	17.4	28.8	33.5		
	*p*-value	0.144	0.767	0.799	0.162		
Goods coverage	0 d	1.00	1.00	1.00	1.00	–	–
	10 d	1.00	1.00	1.00	1.00	–	–
	SEM	–	–	–	–		
	*p*-value	–	–	–	–		

CK, ensiling *L. chinensis* with 2.00 ml/kg fresh weight (FW) of distilled water; L, ensiling *L. chinensis* with 2.00 g/t FW of lactic acid bacteria (LAB) inoculant and 2.00 ml/kg FW of distilled water; W, ensiling *L. chinensis* with 100 ml/kg FW of distilled water; LW, ensiling *L. chinensis* with 2.00 g/t FW of LAB inoculant and 100.0 ml/kg FW of distilled water. Values with different lowercase letters (a and b) indicate the significant differences among treatments on the same day (*p* < 0.05). Values with different uppercase letters (A and B) indicate the significant differences between 0 and 10 days of opening for the same treatment (*p* < 0.05). SEM, standard error of the means.

**FIGURE 2:**
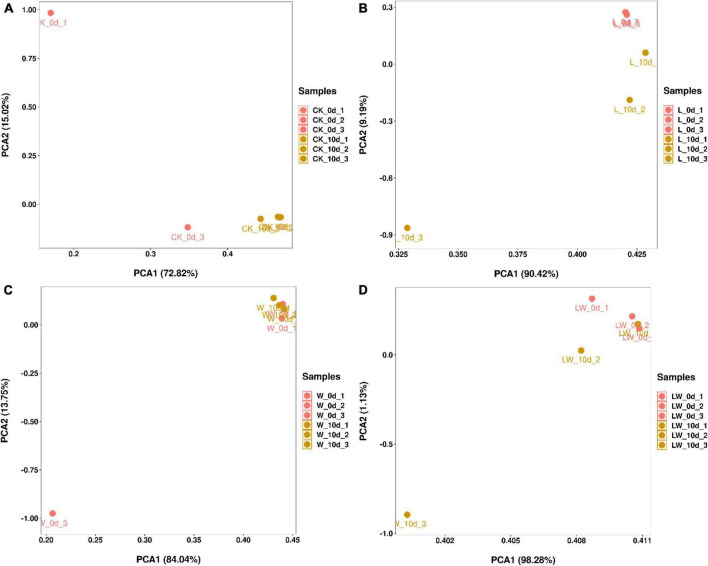
The principal component analysis in *Leymus chinensis* silages (*n* = 3). CK, ensiling *L. chinensis* with 2.00 ml/kg fresh weight (FW) of distilled water **(A)**; L, ensiling *L. chinensis* with 2.00 g/t FW of lactic acid bacteria (LAB) inoculant and 2.00 ml/kg FW of distilled water **(B)**; W, ensiling *L. chinensis* with 100 ml/kg FW of distilled water **(C)**; LW, ensiling *L. chinensis* with 2.00 g/t FW of LAB inoculant and 100.0 ml/kg FW of distilled water **(D)**.

According to PCA, the CK_0d_1 and CK_0d_3 had separated bacterial community from the CK_0d_2 and CK_10d, which had clustered bacterial community (Figure 2). The bacterial community of L_0d was clustered and clearly distinct from L_10d, which had separated bacterial community from each other. The W_0d_3 had separated bacterial community from the W_0d_1, W_0d_2, and W_10d, which had clustered bacterial community. The LW_0d and LW_10d_1 had clustered bacterial community, but the LW_10d had separated bacterial community.

### Bacterial community

**FIGURE 3:**
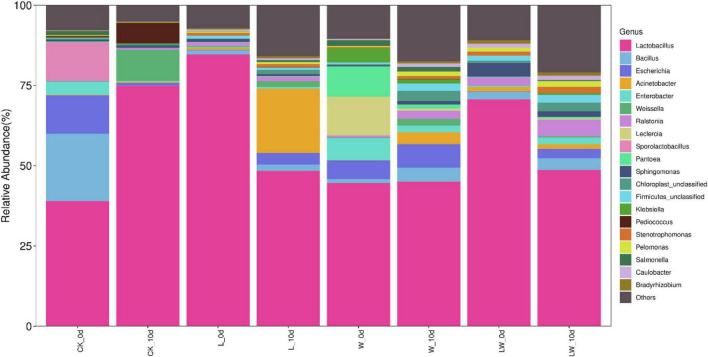
The relative abundance of bacterial community (genus level) in *Leymus chinensis* silages at 0 and 10 days of opening (*n* = 3). CK, ensiling *L. chinensis* with 2.00 ml/kg fresh weight (FW) of distilled water; L, ensiling *L. chinensis* with 2.00 g/t FW of lactic acid bacteria (LAB) inoculant and 2.00 ml/kg FW of distilled water; W, ensiling *L. chinensis* with 100 ml/kg FW of distilled water; LW, ensiling *L. chinensis* with 2.00 g/t FW of LAB inoculant and 100.0 ml/kg FW of distilled water.

*Lactobacillus* was the most bacterial genus in CK_0d, L_0d, W_0d, and LW_0d, with the abundance of 39.0, 84.6, 44.7, and 70.6%, respectively, followed by *Bacillus* (20.9%), *Escherichia* (12.0%), and *Sporolactobacillus* (11.9%) in CK_0d, and *Leclercia* (12.1%), *Pantoea* (9.50%), *Enterobacter* (6.78%), and *Escherichia* (5.73%) in W_0d (Figure 3). At 10 days of opening, the abundance of *Lactobacillus* increased to 74.9% in CK_10d and to 45.1% in W_10d, respectively, and reduced to 48.4% in L_10d and to 48.7% in LW_10d, respectively. *Bacillus*, *Escherichia*, and *Sporolactobacillus* reduced to as minor taxa in CK_10d, with the abundance of 0.12, 0.88, and 0%, respectively. The abundances of *Escherichia* and *Acinetobacter* in L_10d increased to 3.59 and 20.1%, respectively. In W_10d, the abundances of *Leclercia*, *Pantoea*, and *Enterobacter* decreased to 0.69, 1.31, and 2.05%, respectively; however, *Escherichia* and Escherichia increased to 7.37 and 4.29%, respectively. In LW_10d, the *Bacillu*s and *Ralstonia* increased to 3.67 and 5.15% from 2.44 to 2.50% at 0 day, respectively, whereas *Sphingomonas* decreased to 1.78% from 4.36% at 0 day.

**FIGURE 4:**
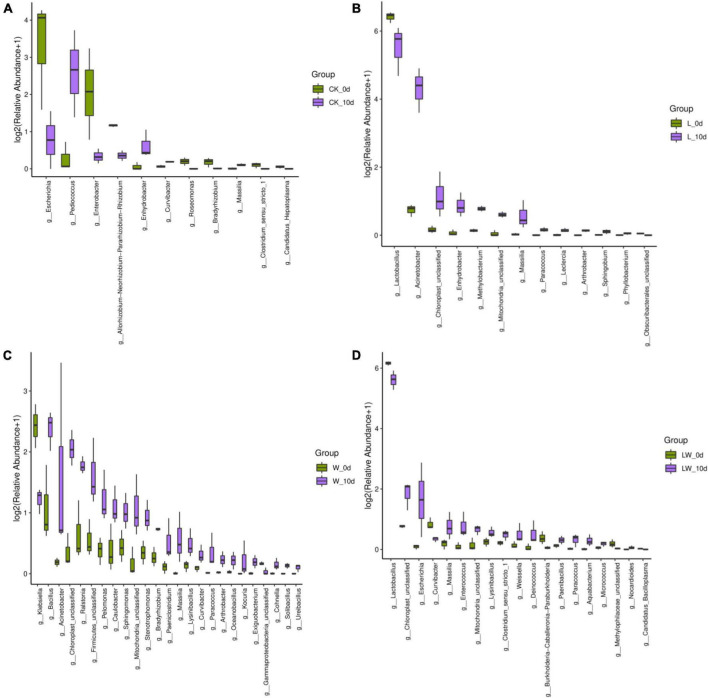
Difference in bacterial communities (genus level) in *Leymus chinensis* silages between 0 and 10 days of opening for each treatment (*n* = 3). CK **(A)**, ensiling *L. chinensis* with 2.00 ml/kg fresh weight (FW) of distilled water; L **(B)**, ensiling *L. chinensis* with 2.00 g/t FW of lactic acid bacteria (LAB) inoculant and 2.00 ml/kg FW of distilled water; W **(C)**, ensiling *L. chinensis* with 100 ml/kg FW of distilled water; LW **(D)**, ensiling *L. chinensis* with 2.00 g/t FW of LAB inoculant and 100.0 ml/kg FW of distilled water.

The CK_10 d contained higher *Pediococcus*, but lower *Escherichia* and *Enterobacter* than CK_0 d (*p* < 0.05) (Figure 4). The L_10d contained higher *Acinetobacter* and *Enhydrobacter*, whereas lower *Lactobacillus* than L_0 d (*p* < 0.05). The W_10 d contained higher *Acinetobacter* and *Ralstonia*, and lower *Klebsiella* and *Bacillus* than W_0 d (*p* < 0.05). The LW_10 d contained higher *Escherichia*, and lower *Lactobacillus* than LW_0 d (*p* < 0.05).

### Nutrition compositions

At 0 and 10 days of opening, the DM content in L contained was higher than that in CK whereas lower than that in W and LW (*p* < 0.01); moreover, at 0 day of opening, the ash concentration in LW was higher than that in CK but lower than that in W (*p* < 0.01) (Table 5). The CP concentrations in L_10d and W_10d were lower than that in L_0d and W_0d, respectively (*p* < 0.05); nevertheless, the NDF and ADF concentrations in L_10d and LW_10d were higher than those in L_0d and LW_0d, respectively (*p* < 0.05).

**TABLE 5:** The dry matter content (DM, g/kg) and nutritional compositions concentrations (g/kg DM) of *Leymus chinensis* silages (*n* = 3).

Items		CK	L	W	LW	SEM	*p*-value
DM	0 d	591a	544b	491c	485c	5.10	<0.001
	10 d	581a	544b	497c	489c	2.46	<0.001
	SEM	4.77	4.82	2.93	3.09		
	*p*-value	0.189	0.963	0.202	0.345		
CP	0 d	10.0	9.47A	9.77A	9.87	0.270	0.522
	10 d	9.21	9.31B	9.35B	9.58	0.150	0.404
	SEM	0.420	0.014	0.073	0.110		
	*p*-value	0.229	0.001	0.015	0.132		
NDF	0 d	351	354B	382	362B	15.3	0.509
	10 d	407	400A	398	414A	14.0	0.845
	SEM	21.6	7.41	15.2	10.6		
	*p*-value	0.140	0.012	0.507	0.025		
ADF	0 d	180	185B	202	186B	7.93	0.297
	10 d	197	209A	203	213A	7.43	0.486
	SEM	12.3	3.53	5.64	6.32		
	*p*-value	0.368	0.008	0.845	0.035		
Ash	0 d	53.1c	56.4ab	57.5a	55.3b	0.430	<0.001
	10 d	54.2	55.9	57.3	56.3	0.756	0.099
	SEM	0.603	0.247	0.381	0.971		
	*p*-value	0.267	0.161	0.729	0.494		

CK, ensiling *L. chinensis* with 2.00 ml/kg fresh weight (FW) of distilled water; L, ensiling *L. chinensis* with 2.00 g/t FW of lactic acid bacteria (LAB) inoculant and 2.00 ml/kg FW of distilled water; W, ensiling *L. chinensis* with 100 ml/kg FW of distilled water; LW, ensiling *L. chinensis* with 2.00 g/t FW of LAB inoculant and 100.0 ml/kg FW of distilled water. CP, crude protein; NDF, neutral detergent fiber; ADF, acid detergent fiber. Values with different lowercase letters (a, b, and c) indicate the significant differences among treatments on the same day (*p* < 0.05). Values with different uppercase letters (A and B) indicate the significant differences between 0 and 10 days of opening for the same treatment (*p* < 0.05). SEM, standard error of the means.

## Discussion

*Leymus chinensis* silage is one of the main forages for ruminants on meadow and typical steppes in Inner Mongolia, Northern China, throughout year. However, the previous studies concerned mainly the short fermentation process of *L. chinensis* silage (less than 100 days) (Xue et al., 2017; Zhang and Yu, 2017; Sun et al., 2020; Xu et al., 2021). In the study, the fermentation quality, bacterial community, and aerobic stability of *L. chinensis* silage after long-term (300 days) fermentation were studied, which can help to provide the high-quality *L. chinensis* silage to ruminants all year-round.

In the study, the scores and marks of all silages were 100 and first, respectively, according to the evaluation system for a fermentation quality based on butyric and acetic acids contents in silage (Kaiser and Weiss, 2005), owing to no butyric acid detected and acetic acid content less than 30 g/kg DM in all silages. Those indicated that the *L. chinensis* silage at 300 days of storage had satisfactory fermentation quality. There were higher AA than LA in CK_0d (14.7 vs. 4.20 g/kg, Table 2), which was in line with the results of previous studies about *L. chinensis* silage (Xue et al., 2017; Xu et al., 2021). Moreover, *Enterobacteriaceae* in CK_0d had 17.7% of abundance and was one of the main dominant families (Supplementary Figure 1). Xu et al. (2021) reported that *Enterobacteriaceae* dominates the bacterial community and utilizes the LA to AA and other products during fermentation (from 5 to 60 days) in *L. chinensis* silage without any treatment. Those resulted in the unique fermentation quality (AA > LA) of CK_0d in the study. Previous studies revealed that low moisture content and insufficient LAB count in the material were the main factors restricting the fermentation of *L. chinensis* silage (Tian et al., 2014; Zhang et al., 2015a; Zhang and Yu, 2017; Xu et al., 2021). In the study, ensiling *L. chinensis* with LAB or/and water improves the fermentation quality of silage, as reflected by the lower pH and higher LA content in L_0d, W_0d, and LW_0d than those in CK_0d (Table 2). The similar results were also detected by Xu et al. (2021) in *L. chinensis* silage. Moreover, You et al. (2021) reported that inoculating LAB can decrease pH and increase LA concentration in native grass silage with low moisture content (407 g/kg). In the study, the W_0d contained higher AN content than other treatments, which might be in connection with a higher abundance of *Enterobacteriaceae* in W_0d (Supplementary Figure 1). *Enterobacteriaceae* has ability to degrade protein to AN in silage during fermentation (Buxton et al., 2003). Moreover, the AN content in all silages was kept low level (from 1.45 to 5.91 g/kg TN), because of the low moisture content (less than 515 g/kg) and the long-term storage (300 days) inhibiting the activity of harmful microbe in *L. chinensis* silages. The bacterial activity during fermentation contributes most strongly to BC of silage and inoculating LBA or/and adding water can make the bacterial more active during early stage of fermentation in *L. chinensis* silage (Xu et al., 2021), which resulted in higher BC in L_0d, W_0d, and LW_0d that in CK_0d in the study. In addition, Xu et al. (2021) found that the volatile components (organic acids and AN) have a declining concentration in *L. chinensis* silage after 35 days of storing. So, in the study, the concentrations of LA, AA, and AN kept in low level (Table 2) and the relevant mechanisms need to be further studied.

The LAB and yeasts were only detected in CK at 0 day of opening, and CK_0d contained the higher aerobic bacteria count than other treatments with pH less than 4.0 and DM content more than 480 g/kg after 300 days of ensiling (Tables 2, 3, 5). Xu et al. (2021) also reported that the LAB, aerobic bacteria, and yeasts counts had a declining trend in *L. chinensis* silage with LAB or/and water after 5 days of storage. However, the LAB and yeasts were detected in whole-plant corn silage at 300 and 350 days of storage with more than 620 g/kg of moisture and less than 3.7 of pH (Bai et al., 2021; Wang et al., 2021). Those indicated that the LAB and yeasts in *L. chinensis* silage became inactivated after long storage (300 days) under low pH (< 4.00) and moisture condition (<515 g/kg).

The previous study reported that *Lactobacillus* has the highest abundance in *L. chinensis* silage at 60 days of storage (Xu et al., 2021); in the study, *Lactobacillus* was the most dominant bacterial genus in all silages (from 39.0 to 84.6%) at 300 days of storage (Figure 3). Those indicated that *Lactobacillus* dominate generally the bacterial community of *L. chinensis* silage from 60 to 300 days of storage. Moreover, *Lactobacillus* dominated the bacterial community of native grass silage with LAB at 30 and 60 days of storage (You et al., 2021; Li et al., 2022). Previous studies reported that *Lactobacillus* was also the main bacterial genus in whole-plant corn silage at 300 days of storage (Wang et al., 2021), but *Acinetobacter* dominated the bacterial community in whole-plant corn silage at 350 days of storage (Bai et al., 2021). At 0 day of opening, the CK had higher of abundance of *Bacillus* than other treatments (Figure 3), owing to the higher pH in CK_0d (Table 2). *Bacillus* likely present in the silage with high pH (Kung et al., 2018). The CK_0d and W_0d contained higher *Escherichia* than L_0d and LW_0d (Supplementary Figure 2); moreover, the similar results were reported in our previous study (Xu et al., 2021). This indicated that inoculating LAB at ensiling *L. chinensis* effectively inhibit *Escherichia*, as potentially pathogenic microorganism, in silage from 60 to 300 days. *Sporolactobacillus*, as one of the LAB genera, was detected in whole-plant corn silage for the first time (Kharazian et al., 2017; Hu et al., 2018). It is a facultative anaerobic, spore-forming, and gram-positive bacterium and can convert sugar to D-lactic acid (Yanagida and Suzuki, 2015). In the study, *Sporolactobacillus* was one of the main bacterial genera (11.9%) in CK_0d (Figure 3). Although *Sporolactobacillus* has antifungal activities in whole-plant corn silage with from 30 to 40% of DM (Kharazian et al., 2017), yeasts were detected in CK_0d with 60% of DM. The reason might be that the lower moisture in *L. chinensis* silage limits the ability to inhibit fungi. *Leclercia* was one of the main bacterial genera (12.1%) in W_0d (Figure 3) and detected in silage for the first time in the study. The W_0d had 3.88 pH and contained 509 g/kg of moisture, which indicated that *Leclercia* has strong resistance to the high acidic and low moisture environment. Moreover, *Leclercia* is probably an opportunistic pathogen that occasionally causes extraintestinal infections in humans (O’Hara and Farmer, 2015) and its effect on silage needs further study.

In the study, during 10 days after opening, CK had 2°C above the ambient temperature (at 224 h of opening), but other treatments had less than 2°C above the ambient temperature (Figure 1). Previous studies reported that the temperature of *L. chinensis* silage after 45 and 90 days of storage was 2°C higher than the ambient temperature at less than 140 h of opening (Zhang et al., 2015a; Sun et al., 2020). Moreover, during aerobic exposure, the coliforms and yeast counts increased in CK, but were not detected in other treatment (Table 3). Those indicated that *L. chinensis* silage after long-term storage (300 days) had greater aerobic stability than that after short-term storage (<100 days), and the coliforms and yeasts contributed to the aerobic deterioration of *L. chinensis* silage after long-term storage. In the study, L_10d, W_10d, and LW_10d had lower pH than L_0d, W_0d, and LW_0d, respectively, although the differences between L_10d and L_0d and LW_10d and LW_0d did not reach the significant level (Table 2). Moreover, the previous studies also reported that the pH reduces or does not change in the first 2 or 3 days of aerobic exposure in *L. chinensis* silage (Zhang et al., 2015a,^2017^), in whole-plant corn silage (Bernardi et al., 2019; Bai et al., 2021; Wang et al., 2021), in alfalfa silage (Yuan et al., 2017), smooth bromegrass silage (Niu et al., 2018), and in Napier grass silage (Mugabe et al., 2020). Those indicated that the pH in silage commonly has a decreasing trend or does not change during initial stage of aerobic exposure. The LA and AA concentrations in all treatments had no difference (except LA in L), but reduced from 0 to 10 days of opening; moreover, the BC in L, W, and LW decreased from 0 to 10 days of opening (Table 2). Those suggested that the reducing BC might contribute mainly to the reducing pH during aerobic exposure in *L. chinensis* silage treated with LAB or/and water in the study. The L_10d, W_10d, and LW_10d contained lower LAB and aerobic bacteria counts than CK_10d; moreover, in L_10d, W_10d, and LW_10d, coliforms and yeasts were not detected, pH was less than 4.0, and moisture content was less than 515 g/kg (Tables 2, 3). It indicated that the acidic and low moisture condition in *L. chinensis* silage treated with LAB or/and water after long-term storage might be the main factors for inhibiting the microbial activity during aerobic exposure. However, previous studies reported that the microbial counts (except coliforms) kept high level during aerobic exposure in whole-plant corn silage after long-term storage (more than 300 days) with pH less than 3.50 and moisture content more than 600 g/kg (Bai et al., 2021; Wang et al., 2021).

In the study, the abundance of *Lactobacillus* in CK increased considerably from 0 to 10 days of opening, without a significant level (Figures 3, 4A), owing to the sample of CK_0d_2 with similar bacterial community with CK_10d (Figure 1A). The rising abundance of *Lactobacillus* during aerobic exposure was also detected in whole-plant corn silage and in sugarcane top silage, with *Lactobacillus* as not the most dominated bacterial genus in silage at opening day (Zhang et al., 2019; Bai et al., 2021). However, *Lactobacillus* in L and LW had a reducing abundance from 0 to 10 days (Figures 3, 4B,D); the similar results were also detected in sugarcane top silage and in barley silages, with *Lactobacillus* as the most dominated bacterial genus in silage at opening day (Liu et al., 2019; Wang et al., 2020). In addition, compared with W_0d, W_10d had no different *Lactobacillus*, lower *Klebsiella*, but higher *Bacillus* and *Acinetobacter* (Figure 4C). The dynamics of microbial communities during aerobic exposure need further study in *L. chinensis* silage after long-term storage (more than 300 days).

## Conclusion

The *L. chinensis* silage after long-term storage (300 days) had satisfactory fermentation quality and aerobic stability. Inoculating LAB and adding water could improve fermentation quality and aerobic stability and increase the abundance of *Lactobacillus* in *L. chinensis* silage. The lower pH and moisture condition were the main factors for inhibiting the microbial activity in *L. chinensis* silage. During aerobic exposure, the reducing BC in *L. chinensis* silage treated with LAB or/and water mainly contributed to the decreasing or no difference in pH; moreover, the abundance of *Lactobacillus* reduced in *L. chinensis* silage treated with LAB or water.

## Data availability statement

The datasets presented in this study can be found in online repositories. The names of the repository/repositories and accession number(s) can be found in the article/Supplementary material.

## Author contributions

HX, YX, and JZ designed the study, wrote the manuscript, and reviewed and edited the manuscript. HX, YX, NN, NW, YZ, LS, MQ, and TW performed the experiments. HX and YX analyzed the data. YX and JZ funded and supervised the experiments. All authors reviewed the manuscript.

## Supplementary material

Supplementary Figure 1 The relative abundance of bacterial community (family level) in *Leymus chinensis* silages at 0 and 10 days of opening (*n* = 3). CK, ensiling *L. chinensis* with 2.00 m/kg fresh weight (FW) of distilled water; L, ensiling *L. chinensis* with 2.00 g/t FW of lactic acid bacteria (LAB) inoculant and 2.00 m/kg FW of distilled water; W, ensiling *L. chinensis* with 100 ml/kg FW of distilled water; LW, ensiling *L. chinensis* with 2.00 g/t FW of LAB inoculant and 100.0 ml/kg FW of distilled water.

Supplementary Figure 2 Difference in bacterial communities (genus level) in *Leymus chinensis* silages among CK, L, W, and LW at 0 day of opening for each treatment (*n* = 3). CK, ensiling *L. chinensis* with 2.00 ml/kg fresh weight (FW) of distilled water; L, ensiling *L. chinensis* with 2.00 g/t FW of lactic acid bacteria (LAB) inoculant and 2.00 ml/kg FW of distilled water; W, ensiling *L. chinensis* with 100 ml/kg FW of distilled water; LW, ensiling *L. chinensis* with 2.00 g/t FW of LAB inoculant and 100.0 ml/kg FW of distilled water.
