# Supplementary figures and images for: Fermentation quality, bacterial community, and aerobic stability of ensiling Leymus chinensis with lactic acid bacteria or/and water after long-term storage
Source: Front Microbiol. 2022 Oct 18;13:959018. doi: 10.3389/fmicb.2022.959018 (PMC9623030; doi:10.3389/fmicb.2022.959018)

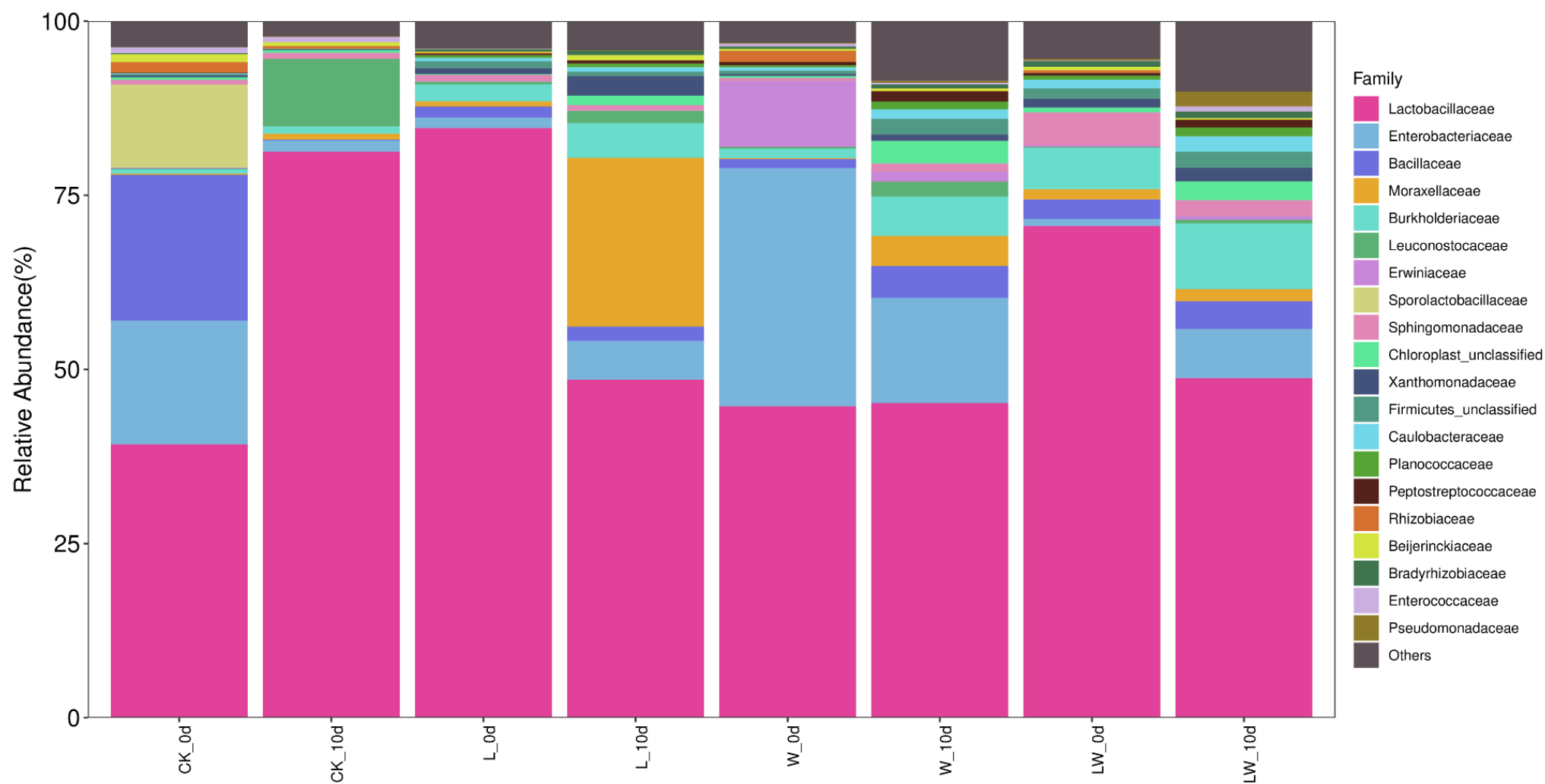

Supplement: Supplementary Figure 1 — The relative abundance of bacterial community (family level) in Leymus chinensis silages at 0 and 10 days of opening (n = 3). CK, ensiling L. chinensis with 2.00 m/kg fresh weight (FW) of distilled water; L, ensiling L. chinensis with 2.00 g/t FW of lactic acid bacteria (LAB) inoculant and 2.00 m/kg FW of distilled water; W, ensiling L. chinensis with 100 ml/kg FW of distilled water; LW, ensiling L. chinensis with 2.00 g/t FW of LAB inoculant and 100.0 ml/kg FW of distilled water. [file Data_Sheet_1.PDF]

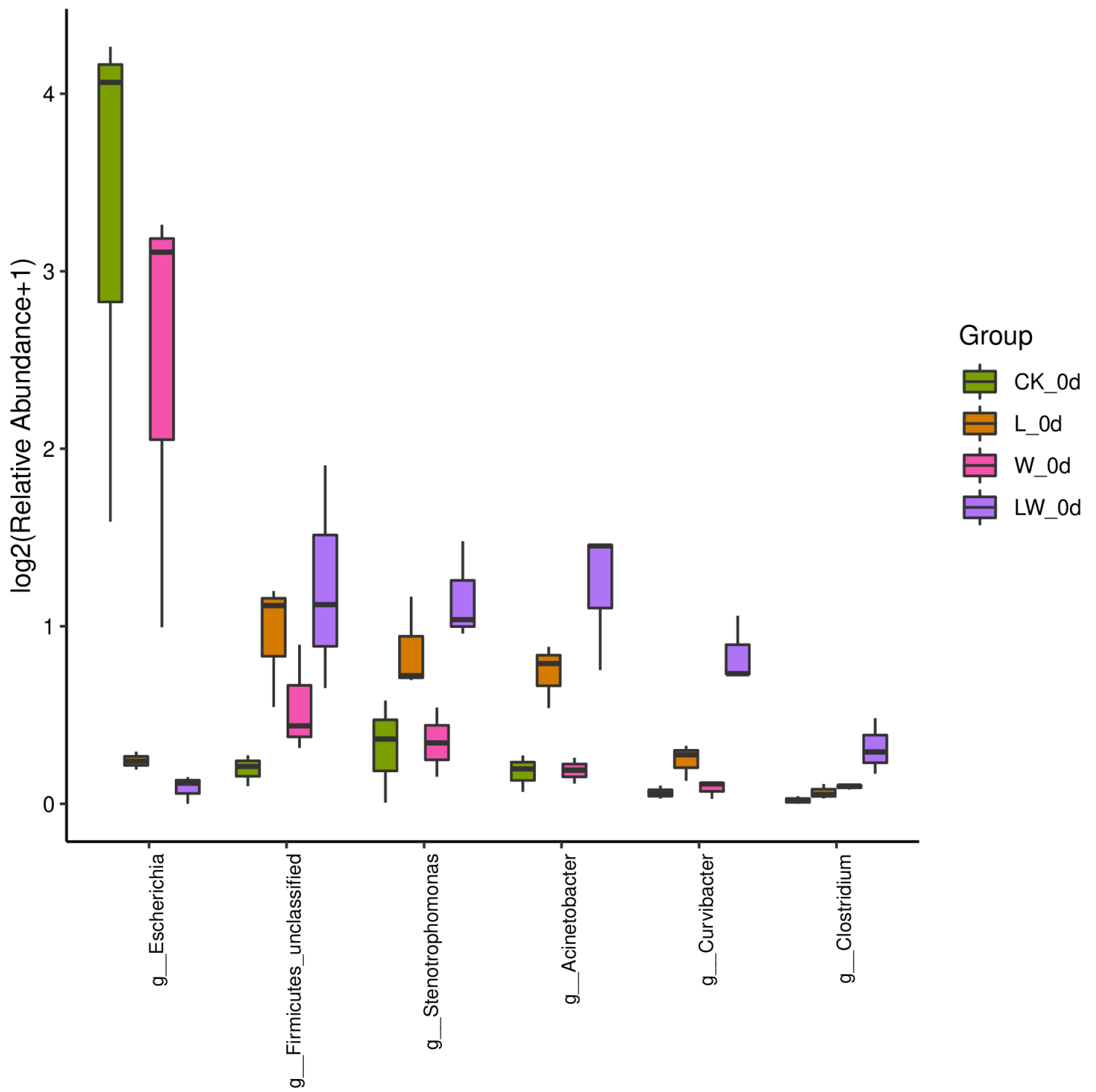

Supplement: Supplementary Figure 2 — Difference in bacterial communities (genus level) in Leymus chinensis silages among CK, L, W, and LW at 0 day of opening for each treatment (n = 3). CK, ensiling L. chinensis with 2.00 ml/kg fresh weight (FW) of distilled water; L, ensiling L. chinensis with 2.00 g/t FW of lactic acid bacteria (LAB) inoculant and 2.00 ml/kg FW of distilled water; W, ensiling L. chinensis with 100 ml/kg FW of distilled water; LW, ensiling L. chinensis with 2.00 g/t FW of LAB inoculant and 100.0 ml/kg FW of distilled water. [file Data_Sheet_2.PDF]
